# Obstetrical outcomes after vaginal repair of caesarean scar diverticula in reproductive-aged women

**DOI:** 10.1186/s12884-018-2015-7

**Published:** 2018-10-19

**Authors:** Xingchen Zhou, Xiaoqian Yang, Huihui Chen, Xuhong Fang, Xipeng Wang

**Affiliations:** 1 Department of Gynecology, Xinhua Hospital affiliated with Shanghai Jiaotong University, 1665 Kong Jiang Rd, Yang Pu District, Shanghai, 200092 China; 2 Department of Gynecology, Shanghai First Maternity and Infant Hospital, Affiliated to Tongji University, Shanghai, China

**Keywords:** Caesarean scar defect, Vaginal repair, Prolonged menstrual bleeding, The thickness of the remaining muscular layer, Obstetrical outcomes

## Abstract

**Background:**

Although vaginal repair has been conducted to manage caesarean scar diverticula, most studies evaluated only the gynaecological outcomes post-surgery, and their obstetrical outcomes were unknown. This study aimed to evaluate the obstetrical outcomes in vaginal repair-treated caesarean scar diverticula patients.

**Methods:**

A series of 51 symptomatic women with caesarean scar defects or a thickness of the remaining muscular layer of less than 3 mm according to transvaginal ultrasound were included. We retrospectively evaluated the gynaecological and obstetrical outcomes after vaginal repair and histologically analysed the defect.

**Results:**

Transvaginal ultrasound revealed that the thickness of the remaining muscular layer significantly increased from 2.24 ± 0.81 mm to 6.10 ± 1.43 mm 3 months after vaginal repair. The duration of menstruation significantly decreased from 14.29 ± 3.13 days to 8.31 ± 2.14 days post-vaginal repair. Notably, 26 of the 51 (50.98%) women who were followed for more than 15.04 months post-surgery achieved pregnancy. A total of 6 of the 26 pregnancies (23.08%) resulted in miscarriages, including 5 early miscarriages and 1 late miscarriage. Among the 20 women who achieved pregnancy without miscarriage, 18 had term deliveries, 2 had preterm birth, and none reported uterine rupture. Histological analysis was performed in all 51 cases. Muscle fibre density was significantly lower in the scar than in the myometrium adjacent to the scar and collagen expression was markedly increased in the scar tissue.

**Conclusion:**

Satisfactory gynaecological and subsequent obstetrical outcomes can be achieved in vaginal repair-treated caesarean scar diverticula patients.

## Background

**Fig. 1:**
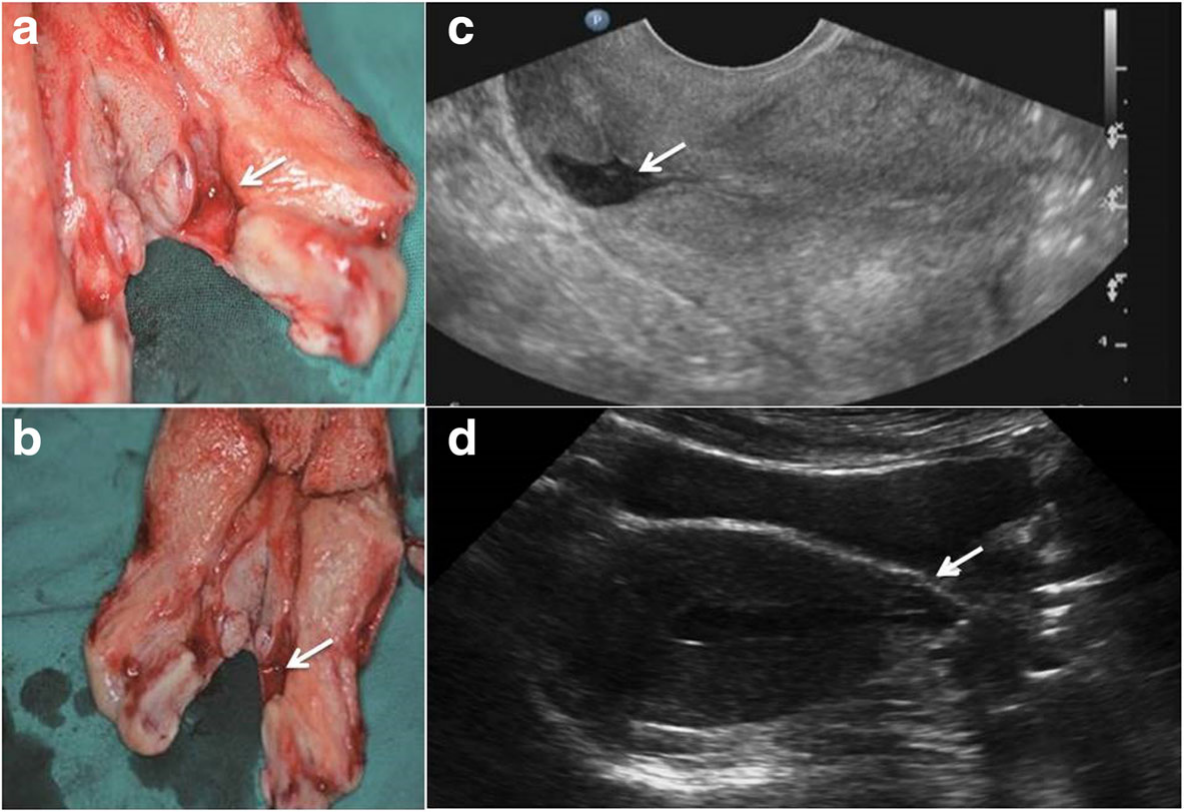
The shape of CSD. **a**, **b**: A section view of a hysterectomy specimen. A deep anterior defect covered with a thin layer of myometrium (white arrow) can be observed at the level of the supposed site of CS. **c, d**: The white arrows indicate the shape of CSD under transabdominal and transvaginal ultrasonography

In China, the proportion of caesarean sections (CSs) performed in 2010 was 35–58%, which has attracted significant concern regarding the development of caesarean scar diverticula (CSD) [1]. CSD are reservoir-like pouch defects at the site of a previous caesarean incision and result from incomplete scar healing on the anterior wall of the uterine isthmus (Fig. 1) [2]. It has been suggested that the incidence of CSD is as high as 61% after one CS and reaches 100% after at least three CSs [3]. With the increasing CS rate and the two-child policy implemented in China, the complications of CSD, such as prolonged menstrual bleeding, secondary infertility, and even uterine rupture during a subsequent pregnancy, have emerged as important clinical problems [4]. The prevalence of CSD-associated prolonged menstrual bleeding ranges from 63.6 to 88% [5]. The exact pathogenesis responsible for CSD has not been fully elucidated. Multiple CS procedures, a retroflexed uterus, cervical dilation, the type and technique of uterine closure, and maternal age at the time of caesarean section were suggested to contribute to the potential risk for CSD [2, 6–8].

Clinical guidelines have yet to be established for the management of CSD. CSD can be treated using a hysteroscopy transcervical approach, laparoscopic abdominal approach, and transvaginal surgical repair. Above all, vaginal repair has been identified by our group and other researchers as a less invasive, safe and effective surgical procedure to treat CSD [9–11]. Vaginal repair can not only resolve CSD-associated prolonged menstrual bleeding but can also restore the anatomical structure in patients with CSD. A retrospective review reported that the improvement in menstruation can reach 93.5% (43/46) in CSD patients who received vaginal repair [9]. Since April 2013, our team has performed vaginal repair to treat CSD. Our previous studies reported that 80.3% (94/117) of CSD patients experienced a duration of menstruation ≤10 days, and the thickness of the remaining muscular layer (TRM) exhibited significant improvements (2.56 ± 1.32 vs 8.65 ± 3.11, *P* < 0.001) at 6 months post-surgery [10]. The results of our other study revealed that 60.0% (48/80) of the patients with CSD that resolved after surgery achieved a duration of menstruation ≤7 days at the median follow-up (11.28 months) [11]. Therefore, we believe that vaginal repair can benefit women who experienced CSD and want to achieve pregnancy again.

However, the subsequent pregnancy outcome in vaginal repair-treated women was not included in our previous studies. To address this deficiency, women who had undergone vaginal repair of CSD, whose TRM was less than 3 mm and who attempted pregnancy were followed long term in our institution. Their clinical characteristics were analysed, and obstetrical outcomes were recorded.

## Methods

### Patient population

A total of 340 CSD patients were managed using vaginal repair between April 2013 and May 2016 at Shanghai First Maternity and Infant Hospital, Tongji University. he presence of remaining myometrium (TRM) was determined by transvaginal ultrasound (TVU), which was clearly described in our previous studies [10, 11] To measure the thickness of the residual muscle layer of the diverticulum, the patient’s should be a cursor was placed at the interface between the uterine and the bladder wall, and another cursor was positioned at the bottom of the CSD. Three different measurements of length, width, height, depth and TRM were obtained. All of the patients underwent TVU to evaluate the parameters of the CSD preoperatively and postoperatively, namely, CSD length, width, depth and TRM. Among the enrolled participants, 51 patients attempted to become pregnant; their clinical characteristics are summarized in Table 1. Our present retrospective study was approved by the Ethics Committee of Shanghai First Maternity and Infant Hospital (KS1512). Informed written consent was obtained from all participants after they received clear information about the procedure and possible surgical outcomes of vaginal repair.

**Table 1:** Clinical characteristics of the patients with CSD

Characteristic	CSD patients (*n* = 51)
Age, y	31.25 ± 3.36 (24–42)
Menstruation, d	
Before VR	14.29 ± 3.13 (8–25)
After VR	8.31 ± 2.14 (5–14)
Uterus position, %	
Anteflexion	37.3% (19/51)
Retroflexion	62.7% (32/51)
Number of previous caesarean sections	1.04 ± 0.20
One, %	96.1% (49/51)
More than one, %	3.9% (2/51)
CSD parameters before VR, mm	
CSD length	7.77 ± 3.39 (2–16)
CSD width	12.00 ± 5.42 (4–27)
CSD depth	6.32 ± 2.45 (2–12)
TRM, mm	2.24 ± 0.81 (0.7–4.6)
Postoperative TRM, mm	8.36 ± 2.96 (3.0–13.9)
CSD resolved after VR, %	68.63% (35/51)
Persistent CSD after VR, %	31.37% (16/51)
Persistent CSD parameters, mm	
CSD length	5.78 ± 2.64 (2–11)
CSD width	9.00 ± 5.32 (3–20)
CSD depth	4.33 ± 1.00 (3–6)
TRM, mm	6.10 ± 1.43 (3.8–8.1)
Pregnancy rate, %	50.98% (26/51)
Spontaneous abortion rate, %	23.08% (6/26)
Median follow-up time, months	28.42 ± 9.74 (15.04–50.46)

Data are presented as the means ± SD or percentages

*Note:* CSD = caesarean scar diverticula; VR = vaginal repair of CSD; TRM = thickness of the remaining muscular layer

### Surgical technique

The complete surgical technique was described in detail in our previous studies and has been recognized as an effective approach for anatomic correction of CSD as well as symptomatic relief of prolonged menstrual duration [11]. The most important steps of vaginal repair of CSD are summarized again here. Briefly, the bladder was carefully dissected away from the uterus towards the abdominal cavity to open the vesicovaginal space and reach the peritoneum. CSD tissue was easily found once the abdominal cavity was entered, and the lower uterine segment was completely exposed. The CSD tissue was then thoroughly removed. All surgical procedures reported in the current series were performed by the same surgeon. All patients were discharged from the hospital on postoperative day 3. After the subsequent TVU at 3 months post-surgery, the women were told they could attempt pregnancy starting 1 year post-surgery if their TRM had reached a thickness of more than 3 mm.

### Data collection and follow-up

Preoperative and postoperative clinical information was gathered, including age; uterus position; reproductive history; menstrual duration; CSD length, width, and depth; and TRM. The follow-up was scheduled at 3 months after surgery for all patients, at which time a TVU was performed. The patients with a postoperative TRM more than 3 mm and a subsequent pregnancy attempt were recorded, and long-term follow-up was conducted. Their gestational age, pregnancy complications, interval from vaginal repair to pregnancy, neonatal birth weight, and infant sex were collected.

### Histology

Histology was performed on tissues from all patients, and histochemical and Masson’s staining were performed on the tissues of the 51 patients who attempted to become pregnant post-surgery to evaluate the muscle or collagen density of the remaining myometrium. Two areas were evaluated: the myometrium covering the scar and the myometrium directly adjacent to the scar. Haematoxylin-eosin staining was used for histology. Masson’s staining was used to detect collagen. Panoramic viewer software was applied for histochemical quantification and morphological analysis.

### Statistical analysis

Data are presented as the means ± SD or percentage when appropriate. A paired t-test was used to analyse preoperative and postoperative data. Statistical analysis was performed using SPSS software (version 22, IBM Co., Armonk, NY, USA). Statistical significance was set at *P* < 0.05.

## Results

### Gynaecological outcomes

The background characteristics of the women with CSD who attempted pregnancy after vaginal repair are shown in Table 1. Three months after the surgical procedure, the TRM dramatically (*P* < 0.001) recovered from 2.24 ± 0.81 mm (range, 0.7–4.6) to 8.36 ± 2.96 mm (range, 3.0–13.9) on TVU. The menstrual duration significantly decreased (*P* < 0.001) from 14.29 ± 3.13 days to 8.31 ± 2.14 days 3 months after vaginal repair. CSD disappeared in 68.63% of patients (35/51) at the 3-month follow-up. We present the clinical characteristics of the women with vaginal repair-treated CSD who achieved pregnancy without miscarriage in Table 2. A total of 19 women underwent one CS, and one woman underwent two CSs before vaginal repair of the CSD. The uterine positions were anteflexion in 11 women and retroflexion in 9 women. TVU revealed that the TRM significantly (*P* < 0.001) increased from 2.42 ± 1.04 mm (range, 1.0–4.6) to 8.36 ± 3.11 mm (range, 3.8–12.0) after the vaginal surgical procedure at the 3-month follow-up. The duration of menstruation significantly decreased (*P* < 0.001) from 14.20 ± 2.76 days to 8.00 ± 2.19 days post-vaginal repair. At the 3-month follow-up, 14 of the 20 (70.0%) women had no evidence of CSD as measured by TVU. The interval from vaginal repair to pregnancy was 32.06 ± 10.09 months (range, 16.88–50.36).

**Table 2:** Clinical characteristics of the women who achieved pregnancy without miscarriage

Characteristic	CSD patients (*n* = 20)
Age, y	31.00 ± 3.06 (26–38)
Menstruation, d	
Before VR	14.2 ± 2.76 (8–20)
After VR	8.00 ± 2.19 (5–13)
Uterus position, %	
Anteflexion	55.0% (11/20)
Retroflexion	45.0% (9/20)
Number of C-section deliveries	1.05 ± 0.22
One, %	95.0% (19/20)
More than one, %	5.0% (1/20)
CSD parameters before VR, mm	
CSD length	7.21 ± 3.14 (2–13)
CSD width	11.74 ± 5.61 (4–20)
CSD depth	6.53 ± 2.65 (2–11)
TRM, mm	2.42 ± 1.04 (1.0–4.6)
Postoperative TRM, mm	8.36 ± 3.11 (3.8–12.0)
CSD resolved after VR, %	70.0% (14/20)
Persistent CSD after VR, %	30.0% (6/20)
Persistent CSD parameters, mm	
CSD length	5.5 ± 2.12 (4–7)
CSD width	6.5 ± 4.95 (3–10)
CSD depth	4.5 ± 0.71 (4–5)
TRM, mm	4.95 ± 1.48 (3.9–6.0)
Interval from VR to pregnancy, months	32.06 ± 10.09 (16.88–50.36)
Gestational age (days)	266.03 ± 6.44 (245–273)
Preterm birth rate, %	10% (2/20)
Neonatal birth weight, g	3149.75 ± 308.24 (2500–3850)
Apgar score (5 min)	10
Pregnancy complication rate, %	20.0% (4/20)
Follow-up time, months	32.06 ± 10.09 (16.88–50.36)

Data are presented as the means ± SD or number (percentage)

*Note: CSD* caesarean scar diverticula, *C-section* caesarean section, *VR* vaginal repair of CSD, *TRM* thickness of the remaining muscular layer, *M* male, *F* female

### Obstetrical outcomes

**Fig. 2:**
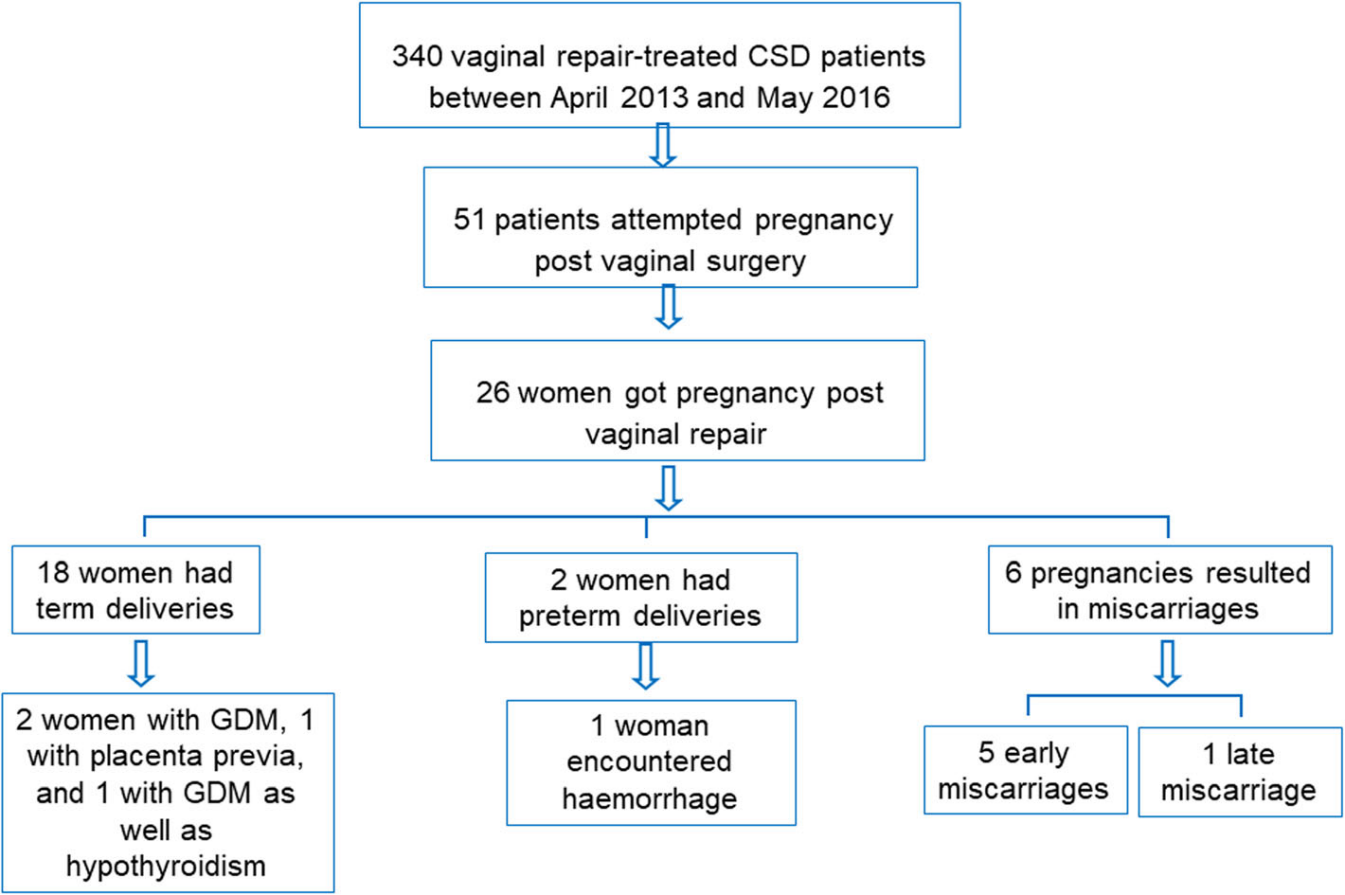
The obstetrical outcomes after vaginal repair of CSD

The obstetrical outcome of pregnancy was assessed in 26 of the 51 women (50.98%) who were followed for more than 15.04 months post-surgery. Unfortunately, 6 of 26 pregnancies (23.08%) resulted in miscarriages, which included 5 early miscarriages and 1 late miscarriage (Tables 1 and 2). None of the women attempted to deliver vaginally, and no cases of threatened uterine rupture or uterine rupture were detected in women who had successful births. As shown in Table 2, the mean gestational age was 266.03 ± 6.44 days, and the mean neonatal birth weight was 3149.75 ± 308.24 g among the women who gave birth (Table 2). The detailed outcomes after vaginal repair of CSD in reproductive-aged women are shown in Table 3. Among the 20 women who achieved pregnancy without miscarriage, 18 had term deliveries, and 2 had preterm deliveries during the long-term follow-up period (32.06 ± 10.09 months). In the women who had preterm deliveries, one woman gave birth at 35 weeks of gestation because of foetal heart rate decelerations on a non-stress test at a routine pregnancy check-up and threatened preterm labour. Another woman gave birth at 36 + 6 weeks of gestation due to preterm premature rupture of the membranes. This woman haemorrhaged 945 ml during the caesarean section. Four women who achieved term deliveries experienced pregnancy complications, including 2 women with gestational diabetes mellitus (GDM), 1 woman with placenta previa, and 1 woman with GDM as well as hypothyroidism during pregnancy (Table 3). However, all 20 women delivered healthy babies (Fig. 2).

**Table 3:** Detailed gynaecological and obstetrical outcomes in vaginal repair-treated CSD patients who achieved pregnancy

Number	Interval form VR to pregnancy, months	Gestational weeks	Preoperative TRM, mm	Postoperative TRM, mm	Preoperative menstruation, d	Postoperative menstruation, d	Time from last cesarean section to menstruation	Neonatal birth weight, g	Apgar score (5 min)	Pregnancy complication	Supplementary information
1	50.36	38 + 5	2.2	6.0	14	12	5	3650	10	GDM, Hypothyroidism in pregnancy	
2	48.06	38	2.0	10.0	15	8	12	2500	10		
3	44.38	38 + 1	1.2	10.5	12	8		3320	10	Placenta previa	Amenorrhoea
4	39.72	37	3.0	10.0	12	7		3000	10		Amenorrhoea
5	39.09	38	3.5	9.5	14	9.5		2945	10	GDM	Amenorrhoea
6	36.50	39	1.6	11.5	15	6	10	3250	10		
7	36.10	38 + 3	2.2	11.0	13	6	13	3200	10		
8	26.22		2.7	10.0	15	7				Spontaneous abortion	
9	35.28	37 + 5	1.4	10.0	20	8	10	3280	10	GDM	
10	34.82	38 + 3	2.9	12.0	17	13	12	2900	10		
11	32.42	38 + 2	1.0	11.0	11	5		3850	10		Amenorrhoea
12	31.96	38	4.5	12.0	15	7.5	7	3500	10		
13	31.83	38	2.0	3.9	14	8.5	7	3150	10		
14	13.34		2.6	10.0	12	6				Spontaneous abortion	
15	31.21	39	2.0	8.0	13	11		3300	10		
16	30.75	38 + 5	2.8	3.8	12	10		3000	10		Amenorrhoea
17	29.70	38 + 6	3.0	4.0	15	8.5		3150	10		Amenorrhoea
18	11.63		1.8	3.0	20	13				Spontaneous abortion	
19	19.64	38 + 4	4.6	9.0	14	7		3200	10		
20	16.39		1.5	6.0	15	8				Spontaneous abortion	
21	18.33	36 + 6	3.1	11.3	15	7		3090	10	preterm premature rupture of the membranes, haemorrhage	Amenorrhoea
22	17.25	38 + 6	1.0	3.9	20	7		3080	10		
23	16.95	35	2.9	5.0	8	5	5	2650	10	Non-reassuring foetal status, Threatened preterm labour	
24	9.10		2.9	4.8	10	4				Spontaneous abortion	
25	16.88	38 + 5	1.5	4.7	15	6		2980	10		
26	3.29		0.7	13.2	17	13				Spontaneous abortion	

*Note: VR* vaginal repair of CSD, *TRM* thickness of the remaining muscular layer, *GDM* gestational diabetes mellitus

### Histology

**Fig. 3:**
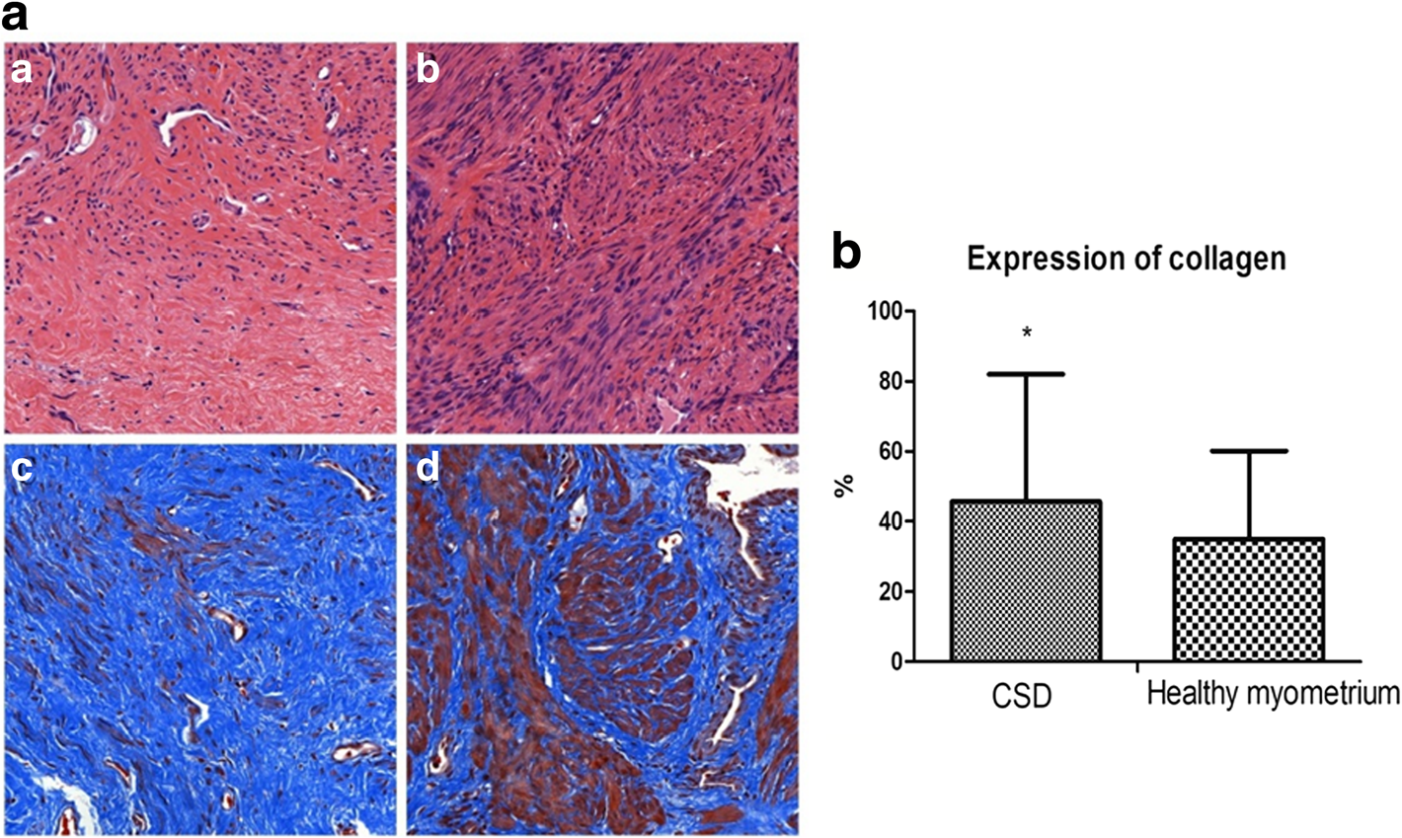
Haematoxylin-eosin staining was used for histology (× 100). **a**: Scar tissue defect. B: Tissue without defect adjacent to the scar; Masson’s staining was used for collagen (× 100). C: Scar tissue defect. **d**: Tissue without defect adjacent to the scar. b: Histogram of the expression of collagen

Histological analysis was performed in all 51 cases. The muscular density of the residual myometrium covering the scar (Fig. 3a and b) was found to be significantly lower (*P* < 0.005) than that directly adjacent to the scar. Moreover, Masson’s staining showed that the expression of collagen in CSD was 71.37 ± 19.82% compared to 52.75 ± 17.33% in healthy myometrium (Fig.3b). The collagen expression significantly increased in the scar defect (Fig. 3c and d).

## Discussion

The most important clinical problems related to CSD are prolonged menstrual bleeding and adverse events that may affect subsequent pregnancies, including infertility, miscarriage, and uterine rupture. The prevalence of CSD-associated prolonged menstrual bleeding ranges from 63.6 to 88% [5]. Although the risk with CSD remains unclear, there is an obvious association between TRM and uterine rupture or dehiscence in subsequent pregnancies [12, 13]. Therefore, repairing the CSD, especially rebuilding the muscular layer of the scar site, might control the symptoms of prolonged menstrual bleeding and decrease the risk of uterine rupture caused by CSD. Hysteroscopy niche resection, laparoscopic repair, laparoscopic-assisted vaginal repair, and vaginal repair are currently employed to treat CSD-related symptoms [1, 14].

Vaginal repair is effective for improving the menstrual duration in patients with CSD. An improvement in uterine bleeding after vaginal repair occurs in 80.3% to 93.5% of cases. A retrospective review reported that the improvements in menstruation can reach 93.5% (43/46) in CSD patients who undergo vaginal repair [9]. Another study revealed that 92.9% (39/42) of vaginal repair-treated CSD patients reported significant improvements in menstruation at follow-up [15]. Our previous studies showed that 80.3% (94/117) of vaginal repair-treated CSD patients experienced a menstrual duration ≤10 days [10]. We also reported that the defect width of the preoperative CSD was the best prognostic index of the CSD anatomical repair effect post-vaginal repair [11]. The prognostic factors for the recovery of normal menstrual duration in CSD vaginal repair-treated CSD patients were assessed in the following study.

Although a series of studies have been conducted to investigate the management of CSD, most of the studies evaluated only the gynaecological outcomes post-surgery, and their obstetrical outcomes were unknown. Moreover, to date, no studies have compared obstetrical outcomes of laparoscopic repair and vaginal repair, and there are no guidelines for the selection of operative strategies for women with CSD who desire to become pregnant post-surgery. It has been reported that pregnancies occurred in 14 of 27 (51.9%) women who underwent laparoscopic surgery [16, 17]. In another study, 10 of 18 (55.6%) women who were treated by laparoscopic repair achieved pregnancy following surgery [18]. The findings from an observational study showed that 44.4% (8/18) of women with infertility conceived and subsequently delivered their new-borns via CS after laparoscopic repair [1]. A total of 50.98% of the women in our study achieved pregnancy after vaginal repair, which indicates that vaginal repair is effective for improving fertility in patients with CSD. CS may lead to reduced fertility and prolonged inter-pregnancy intervals compared with vaginal deliveries. A meta-analysis conducted by Gurol-Urganci et al. suggests that the probability of future pregnancy and birth in women who have had a CS was 9% lower and 11% lower, respectively, than that in women who delivered vaginally [19]. The median inter-pregnancy intervals after CS were 2–6 months longer than those after vaginal delivery [19]. A meta-analysis that included 85,728 women in whom CS was previously performed found the rate of subsequent pregnancy to be reduced by 10% compared with that in women who had vaginal deliveries [20]. Even if the precise pathophysiology remains unclear, incomplete uterine healing and postoperative infection may contribute to the pathophysiological reasons underlying the above phenomenon [21]. The rate of infertility caused by CSD has not been elucidated. It is reasonable to speculate that the rate of infertility in women with CSD was higher than that in women who had undergone CS. The accumulation of mucus or blood in the defect, which leads to the presence of intrauterine fluid, could prevent the penetration of sperm cells, endometrial receptivity, or embryo implantation, which may be responsible for the infertility caused by CSD [1, 18]. In our study, 49.02% of women still did not achieve pregnancy.

Miscarriage was the most common adverse pregnancy outcome in 26 women with recognized pregnancy and previous CSD. Six women (6/26) suffered miscarriage in different stages of pregnancy. The underlying mechanisms of the association between CSD and miscarriage are unclear, and this adverse event may occur with no obvious underlying cause. Some studies report that the rate of spontaneous miscarriage ranges between 10 and 15% of recognized pregnancies [22]. Another study showed that miscarriage occurred in one in five pregnancies in women aged 31–36 years [21]. This number is greater in vaginal repair-treated CSD patients, with a rate of 23.08% reported in the current study. We believe that the miscarriage rate was correlated with age, and the relationship among CSD, miscarriage, and subsequent fertility warrants further investigation.

Uterine rupture is the most feared complication during pregnancy and labour in women who had previous deliveries via CS. TRM in the lower uterine segment (LUS) is a widely accepted indicator for the prediction of uterine rupture during labour. A full thickness of 3.0 mm determined via LUS can be considered a cutoff value to identify women at risk of uterine rupture, with a specificity of 85% and sensitivity of 100% in an observational case-control study [23]. Another study indicated that a full LUS thickness of < 2.3 mm measured between 35 and 38 weeks of gestation is associated with a higher risk of complete uterine rupture [20]. No uterine dehiscence occurred when the full LUS thickness was more than 4.5 mm [24]. In the current study, TRM measured using LUS significantly increased from 2.42 ± 0.1.04 mm to 8.36 ± 3.11 mm, and no cases of threatened uterine rupture or uterine rupture were detected in women who had successful deliveries. This result further demonstrated that vaginal repair has the ability not only to resolve the symptom of prolonged menstrual bleeding but also to rebuild the muscular layer of the scar site.

As reported by Vervoort [25], a number of factors may explain the development of a caesarean scar defect: [1] a very low incision through the cervical tissue; [2] inadequate suturing or incomplete closure of the uterine wall due to a single-layer endometrial-saving closure technique or use of locking sutures; and [3] surgical interventions that encourage adhesion formation (namely, non-closure of the peritoneum, inadequate haemostasis, visible sutures, and others). Our research is the first of its type to detect residual myometrium under the microscope. We found that the collagen density of the residual myometrium covering the scar was significantly higher than that of healthy myometrium directly adjacent to the scar. We believe that the excessive expression of collagen may be an important factor in the formation of diverticula. Thus, the formation of the diverticula may be associated with an imbalance between the ratio of muscle fibres to collagen fibres. Further research will be needed to clarify these issues.

In the current study, 6 of the 20 (30.0%) women who achieved pregnancy without miscarriage experienced pregnancy complications, including 2 women with preterm births, 2 women with GDM, 1 woman with placenta previa, and 1 woman with GDM as well as hypothyroidism during pregnancy. Notably, haemorrhage (945 ml blood) occurred in the patient who had a preterm birth. The ages of the women and the intervals from vaginal repair to pregnancy may be responsible for the relatively high rate of pregnancy complications.

## Conclusion

The current study demonstrated that vaginal repair can not only resolve CSD-associated prolonged menstrual bleeding but can also restore the anatomical structure in patients with CSD. Furthermore, subsequent satisfactory obstetrical outcomes can be achieved in vaginal repair-treated CSD patients.

## Abbreviations

CS: Caesarean section
CSD: Caesarean scar diverticula (defect)
GDM: Gestational diabetes mellitus
LUS: Lower uterine segment
TRM: Thickness of the remaining muscular layer
TVU: Transvaginal ultrasound
